# Bis(2-trifluoro­methyl-1*H*-benzimidazol-3-ium) tetra­chloridomercurate dihydrate

**DOI:** 10.1107/S1600536812018855

**Published:** 2012-05-02

**Authors:** Ming-Liang Liu

**Affiliations:** a Ordered Matter Science Research Center, Southeast University, Nanjing 211189, People’s Republic of China

## Abstract

In the title compound, (C_8_H_6_F_3_N_2_)_2_[HgCl_4_]·2H_2_O, the Hg^II^ cation is coordinated by four Cl^−^ anions in a distorted tetra­hedral geometry. In the crystal, the 2-trifluoro­methyl-1*H*-benzimidazolium cations link to the [HgCl_4_]^2−^ complex anions and lattice water mol­ecules *via* N—H⋯Cl and N—H⋯O hydrogen bonds, and the lattice water mol­ecules further link to the Hg complex anion and the organic cations *via* O—H⋯Cl and O—H⋯F hydrogen bonding. One of the trifluoro­methyl groups is disordered over two orientations in a 0.59 (4):0.41 (4) ratio.

## Related literature

For background to ferroelectric complexes, see: Fu *et al.* (2011 ▶); Ye *et al.* (2009 ▶). Zhang *et al.* (2009 ▶, 2010 ▶, 2012 ▶). For related structures, see: Liu (2011*a* ▶,*b* ▶, 2012*a* ▶,*b* ▶,*c* ▶).
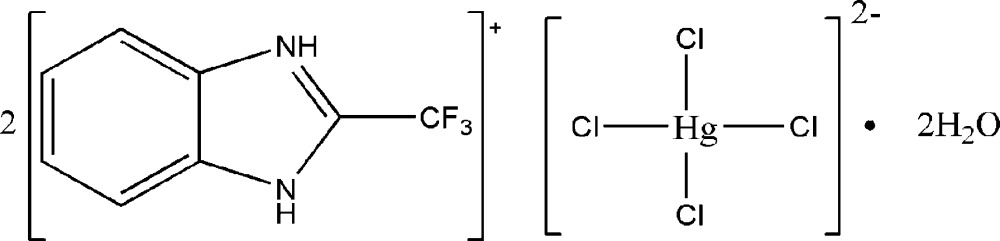


## Experimental

### 

#### Crystal data

(C_8_H_6_F_3_N_2_)_2_[HgCl_4_]·2H_2_O
*M* *_r_* = 752.72
Triclinic, 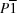

*a* = 9.2485 (18) Å
*b* = 10.029 (2) Å
*c* = 14.754 (3) Å
α = 79.40 (3)°
β = 75.79 (3)°
γ = 67.74 (3)°
*V* = 1221.4 (4) Å^3^
*Z* = 2
Mo *K*α radiation
μ = 6.81 mm^−1^
*T* = 293 K
0.36 × 0.32 × 0.28 mm

#### Data collection

Rigaku SCXmini diffractometer
Absorption correction: multi-scan (*CrystalClear*; Rigaku, 2005 ▶) *T*_min_ = 0.095, *T*_max_ = 0.152
12786 measured reflections
5564 independent reflections
4040 reflections with *I* > 2σ(*I*)
*R*_int_ = 0.061

#### Refinement

*R*[*F* ^2^ > 2σ(*F* ^2^)] = 0.053
*wR*(*F* ^2^) = 0.119
*S* = 1.08
5564 reflections
326 parameters
9 restraints
H-atom parameters constrained
Δρ_max_ = 0.57 e Å^−3^
Δρ_min_ = −1.28 e Å^−3^

### 

Data collection: *CrystalClear* (Rigaku, 2005 ▶); cell refinement: *CrystalClear*; data reduction: *CrystalClear*; program(s) used to solve structure: *SHELXTL* (Sheldrick, 2008 ▶); program(s) used to refine structure: *SHELXTL*; molecular graphics: *SHELXTL*; software used to prepare material for publication: *SHELXTL*.

## Supplementary Material

Crystal structure: contains datablock(s) I, global. DOI: 10.1107/S1600536812018855/xu5519sup1.cif

Structure factors: contains datablock(s) I. DOI: 10.1107/S1600536812018855/xu5519Isup2.hkl

Additional supplementary materials:  crystallographic information; 3D view; checkCIF report

## Figures and Tables

**Table 1:** Hydrogen-bond geometry (Å, °)

*D*—H⋯*A*	*D*—H	H⋯*A*	*D*⋯*A*	*D*—H⋯*A*
N1—H1*A*⋯Cl2^i^	0.86	2.23	3.081 (6)	170
N2—H2*A*⋯O1^ii^	0.86	1.80	2.656 (8)	174
N3—H3*A*⋯O2	0.86	1.76	2.608 (9)	166
N4—H4*A*⋯Cl1	0.86	2.21	3.069 (6)	175
O1—H1*C*⋯F3^iii^	0.85	2.25	2.994 (18)	146
O1—H1*B*⋯Cl2^iv^	0.85	2.55	3.279 (6)	144
O2—H2*B*⋯F5^v^	0.85	2.41	3.224 (13)	160
O2—H2*D*⋯Cl3^vi^	0.85	2.50	3.339 (10)	172

Symmetry codes: (i) 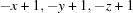; (ii) 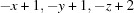; (iii) 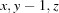; (iv) 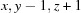; (v) 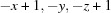; (vi) 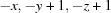.

**Fig. 1.**
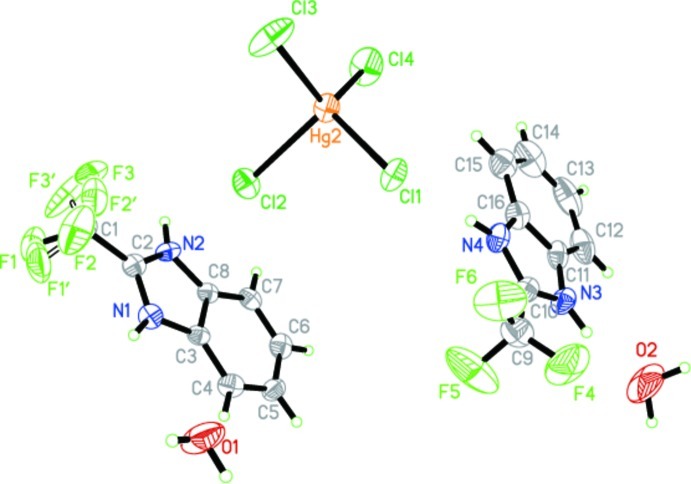
The molecular structure of the title compound, showing the atomic numbering scheme with 30% probability displacement ellipsoids.

**Fig. 2.**
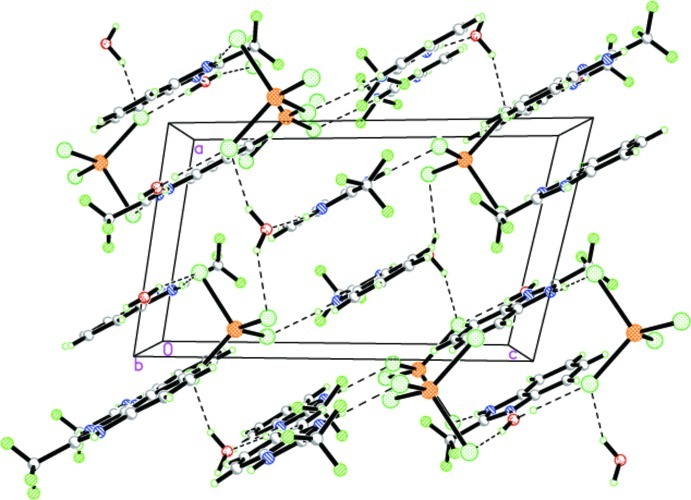
The packing of the title compound with view along the b axis. For the sake of clarity only the major component of the disordered trifluoromethyl group is shown.

## supplementary crystallographic information

### Comment

Recently much attention has been devoted to simple molecular-ionic compounds containing inorganic and organic ions due to the tunability of their special structural features and their potential ferroelectrics property. Ferroelectric materials that exhibit reversible electric polarization in response to an external electric field have found many applications such as nonvolatile memory storage, electronics and optics. The freezing of a certain functional group at low temperature forces significant orientational motions of the guest molecules and thus induces the formation of the ferroelectric phase. (Fu *et al*, 2011; Ye *et al.* 2009; Zhang *et al.* 2009; Zhang *et al.* 2012; Zhang *et al.* 2010). In our laboratory, the title compound has been synthesized to investigate to its potentialferroelectric properties. However, it was found that the dielectric constant of the compound as a function of temperature indicates that the permittivity is basically temperature-independent (*ε* = C/(T–T_0_)), suggesting that this compound is not ferroelectric or there may be no distinct phase transition occurring within the measured temperature (below the melting point).

The title compound,(C_8_H_6_F_3_N_2_)_2_^+^.HgCl_4_^2-^.2H_2_O, has an asymmetric unit that consists of two 2-trifluoromethyl-1*H*-benzimidazol cations, one tetrachloridomercuriate anion and two water molecules (Fig 1). The atoms of the benzimidazole ring are nearly coplanar and the triflouromethyl group lies out of this plane. The mercury cation is coordinated by six Cl^-^ anions in distorted tetrahedral geometry.the average Hg—Cl bond distances range from 2.364 (2) Å to 2.564 (2) Å, the Cl—Hg—Cl angles range from 102.24 (8)°to 120.36 (9)°. In the crystal structure, the 2-trifluoromethyl-1*H*-benzimidazolecations are linked to adjacent tetrachloridomercuriate anions and watermolecules by N—H···O, N—H···Cl and O—H···Cl hydrogen bonds to form one dimensional chains parallel to *ac* plane (Fig 2). One of the trifluoromethyl is disordered.

### Experimental

0.144 g (1 mmol) of 2-trifluoromethyl-1*H*-benzimidazol was firstly dissolved in 30 ml of ethanol which was added hydrochloric acid, to which 0.271 g (1 mmol) of mercuric chloride was added to give a solution at the ambient temperature. Single crystals suitable for X-ray structure analysis were obtained by the slow evaporation of the above solution after 5 days in air.

### Refinement

H atoms were placed in calculated positions (O—H = 0.85 Å; N—H = 0.89 Å; C—H = 0.93Å for C*sp^2^* atoms and C—H = 0.96Å and 0.97Å for C*sp^3^* atoms), assigned fixed *U*_iso_ values [*U*_iso_ = 1.2*U*eq(C*sp^2^*) and 1.5*U*eq(C*sp^3^*,*N*,O)] and allowed to ride. The trifluoromethyl group is disordered over two sites. The site occupancies were refined and restraints were applied to the thermal parameters.

### Crystal data

**Table:**

(C_8_H_6_F_3_N_2_)_2_[HgCl_4_]·2H_2_O	*Z* = 2
*M**_r_* = 752.72	*F*(000) = 716
Triclinic, *P*1	*D*_x_ = 2.047 Mg m^−^^3^
Hall symbol: -P 1	Mo *K*α radiation, λ = 0.71073 Å
*a* = 9.2485 (18) Å	Cell parameters from 4366 reflections
*b* = 10.029 (2) Å	θ = 3.0–26.0°
*c* = 14.754 (3) Å	µ = 6.81 mm^−^^1^
α = 79.40 (3)°	*T* = 293 K
β = 75.79 (3)°	Block, colourless
γ = 67.74 (3)°	0.36 × 0.32 × 0.28 mm
*V* = 1221.4 (4) Å^3^	

### Data collection

**Table:**

Rigaku SCXmini diffractometer	5564 independent reflections
Radiation source: fine-focus sealed tube	4040 reflections with *I* > 2σ(*I*)
Graphite monochromator	*R*_int_ = 0.061
CCD_Profile_fitting scans	θ_max_ = 27.5°, θ_min_ = 3.1°
Absorption correction: multi-scan (*CrystalClear*; Rigaku, 2005)	*h* = −12→12
*T*_min_ = 0.095, *T*_max_ = 0.152	*k* = −13→12
12786 measured reflections	*l* = −19→19

### Refinement

**Table:**

Refinement on *F*^2^	Primary atom site location: structure-invariant direct methods
Least-squares matrix: full	Secondary atom site location: difference Fourier map
*R*[*F*^2^ > 2σ(*F*^2^)] = 0.053	Hydrogen site location: inferred from neighbouring sites
*wR*(*F*^2^) = 0.119	H-atom parameters constrained
*S* = 1.08	*w* = 1/[σ^2^(*F*_o_^2^) + (0.0373*P*)^2^ + 1.355*P*] where *P* = (*F*_o_^2^ + 2*F*_c_^2^)/3
5564 reflections	(Δ/σ)_max_ = 0.022
326 parameters	Δρ_max_ = 0.57 e Å^−^^3^
9 restraints	Δρ_min_ = −1.28 e Å^−^^3^

### Special details

**Table:**

Geometry. All esds (except the esd in the dihedral angle between two l.s. planes) are estimated using the full covariance matrix. The cell esds are taken into account individually in the estimation of esds in distances, angles and torsion angles; correlations between esds in cell parameters are only used when they are defined by crystal symmetry. An approximate (isotropic) treatment of cell esds is used for estimating esds involving l.s. planes.
Refinement. Refinement of F^2^ against ALL reflections. The weighted R-factor wR and goodness of fit S are based on F^2^, conventional R-factors R are based on F, with F set to zero for negative F^2^. The threshold expression of F^2^ > 2sigma(F^2^) is used only for calculating R-factors(gt) etc. and is not relevant to the choice of reflections for refinement. R-factors based on F^2^ are statistically about twice as large as those based on F, and R- factors based on ALL data will be even larger.

### Fractional atomic coordinates and isotropic or equivalent isotropic displacement parameters (Å^2^)

**Table:**

	*x*	*y*	*z*	*U*_iso_*/*U*_eq_	Occ. (<1)
F1	0.7017 (13)	0.721 (2)	0.7840 (6)	0.093 (6)	0.59 (4)
F2	0.499 (3)	0.677 (2)	0.8538 (16)	0.123 (10)	0.59 (4)
F3	0.535 (3)	0.8571 (9)	0.8821 (10)	0.112 (9)	0.59 (4)
F1'	0.656 (4)	0.652 (3)	0.7911 (14)	0.137 (16)	0.41 (4)
F3'	0.639 (4)	0.841 (2)	0.843 (3)	0.16 (2)	0.41 (4)
F2'	0.4537 (9)	0.751 (3)	0.8841 (12)	0.102 (11)	0.41 (4)
N1	0.7197 (6)	0.4910 (6)	0.9609 (4)	0.0463 (14)	
H1A	0.6964	0.4371	0.9319	0.056*	
N2	0.7409 (6)	0.6807 (6)	0.9988 (4)	0.0446 (13)	
H2A	0.7328	0.7692	0.9978	0.053*	
C1	0.6080 (8)	0.7210 (7)	0.8664 (5)	0.064 (2)	
C2	0.6894 (8)	0.6313 (8)	0.9408 (5)	0.0446 (16)	
C3	0.7956 (7)	0.4460 (7)	1.0370 (5)	0.0392 (15)	
C4	0.8554 (9)	0.3110 (8)	1.0833 (5)	0.0522 (18)	
H4	0.8479	0.2294	1.0662	0.063*	
C5	0.9278 (9)	0.3035 (8)	1.1571 (5)	0.0532 (18)	
H5	0.9701	0.2150	1.1913	0.064*	
C6	0.9371 (8)	0.4285 (9)	1.1801 (5)	0.0535 (18)	
H6	0.9860	0.4200	1.2300	0.064*	
C7	0.8793 (8)	0.5611 (8)	1.1340 (5)	0.0456 (16)	
H7	0.8871	0.6425	1.1511	0.055*	
C8	0.8083 (7)	0.5692 (7)	1.0604 (5)	0.0376 (14)	
F4	0.2353 (11)	0.1121 (7)	0.5538 (6)	0.146 (3)	
F5	0.3648 (10)	0.1573 (10)	0.4248 (7)	0.167 (4)	
F6	0.1251 (8)	0.2625 (7)	0.4538 (6)	0.124 (2)	
N3	0.3498 (7)	0.3080 (7)	0.5958 (4)	0.0579 (16)	
H3A	0.3878	0.2271	0.6286	0.070*	
N4	0.2323 (7)	0.4628 (7)	0.4921 (4)	0.0526 (15)	
H4A	0.1822	0.4993	0.4462	0.063*	
C9	0.2500 (13)	0.2136 (11)	0.4902 (7)	0.076 (3)	
C10	0.2768 (8)	0.3277 (8)	0.5263 (5)	0.0503 (17)	
C11	0.3561 (8)	0.4384 (9)	0.6076 (5)	0.0516 (18)	
C12	0.4205 (10)	0.4786 (14)	0.6680 (6)	0.079 (3)	
H12	0.4753	0.4119	0.7117	0.095*	
C13	0.3997 (13)	0.6218 (17)	0.6601 (8)	0.097 (4)	
H13	0.4396	0.6534	0.7008	0.116*	
C14	0.3236 (14)	0.7196 (14)	0.5960 (9)	0.098 (4)	
H14	0.3153	0.8155	0.5935	0.117*	
C15	0.2581 (11)	0.6842 (10)	0.5344 (7)	0.072 (2)	
H15	0.2037	0.7525	0.4912	0.086*	
C16	0.2792 (9)	0.5380 (9)	0.5415 (5)	0.0524 (18)	
Hg2	0.11556 (4)	0.82505 (3)	0.24321 (2)	0.05631 (13)	
Cl1	0.0685 (3)	0.6016 (2)	0.32320 (13)	0.0606 (5)	
Cl2	0.3673 (3)	0.7252 (2)	0.11954 (14)	0.0653 (5)	
Cl3	−0.1015 (4)	0.9354 (3)	0.1548 (2)	0.1100 (11)	
Cl4	0.1746 (4)	0.9720 (3)	0.32658 (19)	0.1004 (9)	
O1	0.2620 (9)	0.0518 (6)	1.0084 (5)	0.099 (2)	
H1C	0.3067	0.0060	0.9601	0.148*	
H1B	0.2751	−0.0072	1.0576	0.148*	
O2	0.4458 (11)	0.0872 (10)	0.7187 (7)	0.166 (4)	
H2B	0.5002	0.0086	0.6939	0.249*	
H2D	0.3630	0.0783	0.7558	0.249*	

### Atomic displacement parameters (Å^2^)

**Table:**

	*U*^11^	*U*^22^	*U*^33^	*U*^12^	*U*^13^	*U*^23^
F1	0.066 (7)	0.132 (15)	0.054 (7)	−0.020 (8)	−0.006 (5)	0.016 (7)
F2	0.085 (14)	0.187 (19)	0.124 (17)	−0.076 (13)	−0.071 (14)	0.047 (14)
F3	0.130 (16)	0.066 (9)	0.099 (9)	0.029 (8)	−0.054 (10)	0.000 (7)
F1'	0.11 (3)	0.20 (3)	0.055 (12)	0.015 (19)	−0.030 (13)	−0.034 (13)
F3'	0.15 (3)	0.16 (3)	0.21 (4)	−0.10 (2)	−0.13 (3)	0.13 (3)
F2'	0.041 (8)	0.17 (3)	0.056 (9)	−0.001 (11)	−0.007 (7)	0.001 (12)
N1	0.040 (3)	0.054 (4)	0.053 (3)	−0.023 (3)	−0.011 (3)	−0.007 (3)
N2	0.037 (3)	0.032 (3)	0.061 (4)	−0.011 (2)	−0.009 (3)	0.001 (3)
C1	0.060 (6)	0.069 (6)	0.057 (5)	−0.020 (5)	−0.013 (4)	0.007 (5)
C2	0.031 (4)	0.050 (4)	0.050 (4)	−0.016 (3)	−0.002 (3)	−0.002 (3)
C3	0.031 (3)	0.037 (4)	0.047 (4)	−0.011 (3)	−0.001 (3)	−0.009 (3)
C4	0.046 (4)	0.048 (4)	0.067 (5)	−0.024 (4)	−0.005 (4)	−0.007 (4)
C5	0.053 (5)	0.047 (4)	0.054 (4)	−0.017 (4)	−0.008 (4)	0.004 (4)
C6	0.037 (4)	0.069 (5)	0.054 (4)	−0.022 (4)	−0.002 (3)	−0.005 (4)
C7	0.039 (4)	0.051 (4)	0.050 (4)	−0.019 (3)	−0.002 (3)	−0.016 (3)
C8	0.028 (3)	0.039 (3)	0.045 (4)	−0.014 (3)	0.002 (3)	−0.006 (3)
F4	0.238 (10)	0.076 (4)	0.153 (6)	−0.092 (5)	−0.050 (6)	0.011 (4)
F5	0.130 (6)	0.189 (8)	0.213 (9)	−0.090 (6)	0.056 (6)	−0.144 (7)
F6	0.119 (5)	0.108 (5)	0.188 (7)	−0.048 (4)	−0.078 (5)	−0.035 (5)
N3	0.041 (4)	0.063 (4)	0.059 (4)	−0.012 (3)	−0.014 (3)	0.013 (3)
N4	0.057 (4)	0.065 (4)	0.044 (3)	−0.031 (3)	−0.017 (3)	0.005 (3)
C9	0.078 (7)	0.074 (6)	0.085 (7)	−0.035 (5)	−0.005 (6)	−0.022 (6)
C10	0.037 (4)	0.054 (4)	0.055 (4)	−0.014 (3)	−0.010 (3)	0.003 (4)
C11	0.038 (4)	0.080 (5)	0.040 (4)	−0.027 (4)	−0.006 (3)	0.002 (4)
C12	0.047 (5)	0.137 (10)	0.056 (5)	−0.038 (6)	0.000 (4)	−0.015 (6)
C13	0.080 (8)	0.163 (13)	0.074 (7)	−0.069 (8)	0.011 (6)	−0.055 (8)
C14	0.100 (9)	0.119 (9)	0.101 (8)	−0.078 (8)	0.019 (7)	−0.040 (8)
C15	0.065 (6)	0.079 (6)	0.075 (6)	−0.037 (5)	−0.003 (5)	−0.008 (5)
C16	0.044 (4)	0.067 (5)	0.054 (4)	−0.032 (4)	−0.004 (4)	−0.007 (4)
Hg2	0.0667 (2)	0.0587 (2)	0.05325 (19)	−0.03158 (16)	−0.01580 (15)	−0.00235 (14)
Cl1	0.0810 (14)	0.0633 (12)	0.0535 (11)	−0.0420 (11)	−0.0290 (10)	0.0135 (9)
Cl2	0.0645 (13)	0.0756 (13)	0.0635 (12)	−0.0374 (11)	0.0002 (10)	−0.0144 (11)
Cl3	0.133 (3)	0.0746 (16)	0.155 (3)	−0.0507 (17)	−0.103 (2)	0.0420 (17)
Cl4	0.158 (3)	0.0916 (18)	0.0906 (17)	−0.0746 (19)	−0.0426 (18)	−0.0120 (15)
O1	0.142 (7)	0.050 (3)	0.112 (5)	−0.033 (4)	−0.048 (5)	0.006 (4)
O2	0.122 (7)	0.135 (7)	0.152 (8)	0.018 (6)	−0.038 (6)	0.076 (7)

### Geometric parameters (Å, º)

**Table:**

F1—C1	1.308 (2)	N3—C10	1.308 (9)
F2—C1	1.309 (2)	N3—C11	1.375 (10)
F3—C1	1.309 (2)	N3—H3A	0.8599
F1'—C1	1.309 (2)	N4—C10	1.300 (9)
F3'—C1	1.309 (2)	N4—C16	1.376 (9)
F2'—C1	1.309 (2)	N4—H4A	0.8601
N1—C2	1.317 (9)	C9—C10	1.469 (11)
N1—C3	1.386 (8)	C11—C16	1.377 (10)
N1—H1A	0.8600	C11—C12	1.374 (12)
N2—C2	1.318 (8)	C12—C13	1.361 (15)
N2—C8	1.357 (8)	C12—H12	0.9300
N2—H2A	0.8600	C13—C14	1.347 (16)
C1—C2	1.453 (10)	C13—H13	0.9300
C3—C4	1.371 (9)	C14—C15	1.368 (14)
C3—C8	1.395 (8)	C14—H14	0.9300
C4—C5	1.390 (10)	C15—C16	1.391 (11)
C4—H4	0.9300	C15—H15	0.9300
C5—C6	1.395 (10)	Hg2—Cl4	2.364 (2)
C5—H5	0.9300	Hg2—Cl3	2.455 (3)
C6—C7	1.350 (10)	Hg2—Cl1	2.4734 (19)
C6—H6	0.9300	Hg2—Cl2	2.564 (2)
C7—C8	1.377 (9)	O1—H1C	0.8500
C7—H7	0.9300	O1—H1B	0.8500
F4—C9	1.274 (11)	O2—H2B	0.8500
F5—C9	1.274 (11)	O2—H2D	0.8500
F6—C9	1.281 (11)		
			
C2—N1—C3	107.7 (6)	N2—C8—C3	106.5 (6)
C2—N1—H1A	126.1	C7—C8—C3	120.8 (6)
C3—N1—H1A	126.2	C10—N3—C11	108.6 (6)
C2—N2—C8	108.9 (6)	C10—N3—H3A	125.8
C2—N2—H2A	125.6	C11—N3—H3A	125.7
C8—N2—H2A	125.5	C10—N4—C16	108.4 (6)
F1—C1—F2'	127.4 (10)	C10—N4—H4A	125.9
F1—C1—F3'	68.6 (15)	C16—N4—H4A	125.7
F2'—C1—F3'	109.5 (15)	F4—C9—F5	107.8 (10)
F1—C1—F3	106.1 (10)	F4—C9—F6	107.4 (9)
F2'—C1—F3	70.4 (12)	F5—C9—F6	105.6 (10)
F1—C1—F2	104.5 (11)	F4—C9—C10	112.6 (9)
F3'—C1—F2	135.0 (11)	F5—C9—C10	110.9 (8)
F3—C1—F2	105.9 (12)	F6—C9—C10	112.3 (8)
F2'—C1—F1'	103.1 (16)	N4—C10—N3	110.6 (7)
F3'—C1—F1'	107.4 (18)	N4—C10—C9	124.1 (8)
F3—C1—F1'	134.0 (13)	N3—C10—C9	125.2 (8)
F2—C1—F1'	68.8 (14)	N3—C11—C16	105.9 (6)
F1—C1—C2	113.7 (8)	N3—C11—C12	132.9 (9)
F2'—C1—C2	115.1 (9)	C16—C11—C12	121.2 (9)
F3'—C1—C2	111.0 (9)	C13—C12—C11	116.1 (10)
F3—C1—C2	113.6 (7)	C13—C12—H12	121.9
F2—C1—C2	112.1 (7)	C11—C12—H12	121.9
F1'—C1—C2	110.1 (9)	C14—C13—C12	122.8 (10)
N1—C2—N2	110.7 (6)	C14—C13—H13	118.6
N1—C2—C1	125.2 (6)	C12—C13—H13	118.6
N2—C2—C1	124.1 (6)	C13—C14—C15	123.0 (11)
C4—C3—N1	130.9 (6)	C13—C14—H14	118.5
C4—C3—C8	122.9 (6)	C15—C14—H14	118.5
N1—C3—C8	106.2 (6)	C14—C15—C16	114.7 (10)
C3—C4—C5	116.0 (6)	C14—C15—H15	122.6
C3—C4—H4	122.0	C16—C15—H15	122.6
C5—C4—H4	122.0	C11—C16—N4	106.5 (7)
C4—C5—C6	120.1 (7)	C11—C16—C15	122.1 (8)
C4—C5—H5	119.9	N4—C16—C15	131.4 (8)
C6—C5—H5	119.9	Cl4—Hg2—Cl3	119.35 (10)
C7—C6—C5	123.8 (7)	Cl4—Hg2—Cl1	120.36 (8)
C7—C6—H6	118.1	Cl3—Hg2—Cl1	102.26 (8)
C5—C6—H6	118.1	Cl4—Hg2—Cl2	105.49 (10)
C6—C7—C8	116.4 (6)	Cl3—Hg2—Cl2	105.13 (11)
C6—C7—H7	121.8	Cl1—Hg2—Cl2	102.10 (8)
C8—C7—H7	121.8	H1C—O1—H1B	109.5
N2—C8—C7	132.7 (6)	H2B—O2—H2D	109.5
			
C3—N1—C2—N2	0.2 (8)	N1—C3—C8—N2	0.0 (7)
C3—N1—C2—C1	−178.8 (6)	C4—C3—C8—C7	−1.7 (10)
C8—N2—C2—N1	−0.2 (8)	N1—C3—C8—C7	−179.9 (6)
C8—N2—C2—C1	178.9 (6)	C16—N4—C10—N3	0.3 (8)
F1—C1—C2—N1	−84.6 (15)	C16—N4—C10—C9	−178.9 (8)
F2'—C1—C2—N1	75.3 (19)	C11—N3—C10—N4	−0.9 (9)
F3'—C1—C2—N1	−160 (2)	C11—N3—C10—C9	178.3 (8)
F3—C1—C2—N1	153.8 (14)	F4—C9—C10—N4	−150.4 (9)
F2—C1—C2—N1	33.7 (19)	F5—C9—C10—N4	88.8 (12)
F1'—C1—C2—N1	−41 (3)	F6—C9—C10—N4	−29.0 (13)
F1—C1—C2—N2	96.5 (14)	F4—C9—C10—N3	30.4 (13)
F2'—C1—C2—N2	−103.6 (17)	F5—C9—C10—N3	−90.4 (12)
F3'—C1—C2—N2	22 (2)	F6—C9—C10—N3	151.8 (9)
F3—C1—C2—N2	−25.1 (14)	C10—N3—C11—C16	1.1 (8)
F2—C1—C2—N2	−145.2 (16)	C10—N3—C11—C12	−178.6 (8)
F1'—C1—C2—N2	140 (2)	N3—C11—C12—C13	−178.5 (8)
C2—N1—C3—C4	−178.1 (7)	C16—C11—C12—C13	1.8 (12)
C2—N1—C3—C8	−0.1 (7)	C11—C12—C13—C14	−1.4 (15)
N1—C3—C4—C5	178.9 (7)	C12—C13—C14—C15	1.3 (17)
C8—C3—C4—C5	1.3 (10)	C13—C14—C15—C16	−1.4 (15)
C3—C4—C5—C6	−0.3 (10)	N3—C11—C16—N4	−0.9 (8)
C4—C5—C6—C7	−0.2 (11)	C12—C11—C16—N4	178.8 (7)
C5—C6—C7—C8	−0.2 (10)	N3—C11—C16—C15	178.1 (7)
C2—N2—C8—C7	180.0 (7)	C12—C11—C16—C15	−2.2 (12)
C2—N2—C8—C3	0.1 (7)	C10—N4—C16—C11	0.4 (8)
C6—C7—C8—N2	−178.8 (7)	C10—N4—C16—C15	−178.5 (8)
C6—C7—C8—C3	1.1 (9)	C14—C15—C16—C11	1.9 (12)
C4—C3—C8—N2	178.2 (6)	C14—C15—C16—N4	−179.5 (8)

### Hydrogen-bond geometry (Å, º)

**Table:**

*D*—H···*A*	*D*—H	H···*A*	*D*···*A*	*D*—H···*A*
N1—H1*A*···Cl2^i^	0.86	2.23	3.081 (6)	170
N2—H2*A*···O1^ii^	0.86	1.80	2.656 (8)	174
N3—H3*A*···O2	0.86	1.76	2.608 (9)	166
N4—H4*A*···Cl1	0.86	2.21	3.069 (6)	175
O1—H1*C*···F3^iii^	0.85	2.25	2.994 (18)	146
O1—H1*B*···Cl2^iv^	0.85	2.55	3.279 (6)	144
O2—H2*B*···F5^v^	0.85	2.41	3.224 (13)	160
O2—H2*D*···Cl3^vi^	0.85	2.50	3.339 (10)	172

Symmetry codes: (i) −*x*+1, −*y*+1, −*z*+1; (ii) −*x*+1, −*y*+1, −*z*+2; (iii) *x*, *y*−1, *z*; (iv) *x*, *y*−1, *z*+1; (v) −*x*+1, −*y*, −*z*+1; (vi) −*x*, −*y*+1, −*z*+1.
